# Perceived algorithmic evaluation and job satisfaction among older employees: the roles of organizational dehumanization and proactive personality

**DOI:** 10.3389/fpsyg.2026.1874672

**Published:** 2026-07-14

**Authors:** Wenjie Qiu, Lian Cheng, Lihua Yi

**Affiliations:** 1 School of Entrepreneurship, Yiwu Industrial and Commercial College, Jinhua, China; 2 Faculty of Economics and Management, Wenhua College, Wuhan, Hubei, China; 3 Xianda College of Economics and Humanities, Shanghai International Studies University, Shanghai, China

**Keywords:** job satisfaction, older employees, organizational dehumanization, perceived algorithmic evaluation, proactive personality

## Abstract

**Background:**

Algorithmic systems are increasingly used to evaluate employees, yet little is known about how older workers in traditional organizations experience algorithmic performance evaluation.

**Objective:**

To examine whether perceived algorithmic evaluation relates to lower job satisfaction through organizational dehumanization, and whether proactive personality weakens this pathway.

**Methods:**

Three-wave time-lagged survey data from 223 employees aged 45+ in 12 financial-services firms across five regions in China. Moderated mediation model (PROCESS Model 7) tested.

**Results:**

PAE positively associated with dehumanization, which predicted lower job satisfaction. Proactive personality buffered the PAE-dehumanization link. Moderated mediation indicated a less harmful indirect effect when proactive personality was higher.

**Conclusion:**

Organizations should pair algorithmic evaluation with transparent, humanizing management practices, especially for less proactive late-career employees.

## 1 Introduction

Algorithmic systems have become integral to the way organizations monitor performance, allocate work, and evaluate employees (Kellogg et al., 2020; Parent-Rocheleau and Parker, 2022). Although much of the empirical research on this transformation has focused on platform-based gig workers (e.g., Wood et al., 2019; Zhang et al., 2025), traditional, hierarchically structured organizations are also adopting data-driven evaluation systems at an accelerating pace, particularly in industries such as banking and finance, where regulatory technology and key performance indicators have long been institutionalized (Meijerink and Bondarouk, 2023; Parent-Rocheleau and Parker, 2022). For employees in these settings, the question is no longer whether algorithms are part of human resource management (HRM), but how being subject to algorithmic evaluation reshapes their work experience and attitudes toward the organization (Wiener et al., 2021). Indeed, the rapid diffusion of data-driven and generative artificial intelligence into core human-resource functions has intensified scholarly and practitioner debate over its consequences for the employment relationship and for employee well-being (Budhwar et al., 2023).

We focus on perceived algorithmic evaluation (PAE), defined as employees’ subjective appraisal that their work performance is being assessed through real-time, algorithm-driven matching, recording, and rating processes (Zhang et al., 2025). Two empirical gaps in this literature motivate the present study. First, although Zhang et al. (2025) provided a validated PAE scale and demonstrated its positive effects on app-workers’ flow experience and service performance, almost all evidence to date concerns gig workers, whose work designs are gamified and short-term in nature. Whether PAE produces similar or different psychological consequences in traditional organizations, where employees rely on long-term relational contracts and accumulated occupational identity, remains an open question (Parent-Rocheleau and Parker, 2022). Second, the symbolic side of algorithmic evaluation, that is, how it shapes employees’ sense of being recognized as a person rather than a number, has been under-theorized; demand-focused mediators such as workload or burnout cannot fully capture the dignity-related concerns voiced by employees encountering algorithmic HRM (Caesens et al., 2017; Christoff, 2014).

We address these gaps by integrating job demands–resources (JD-R) theory (Bakker and Demerouti, 2017; Schaufeli and Taris, 2014) with research on organizational dehumanization (Bell and Khoury, 2025; Caesens et al., 2017) to model how PAE relates to job satisfaction among older employees in China’s financial industry. From a JD-R framework, PAE constitutes a socio-cognitive job demand that signals continuous, quantified accountability (Parent-Rocheleau and Parker, 2022). When sustained, such demands can activate a health-impairment pathway, in which employees come to feel that the organization values them primarily as instruments of measurable output, an experience captured by organizational dehumanization (Caesens et al., 2017; Raisch and Krakowski, 2021). Dehumanization, in turn, is consistently linked to lower job satisfaction (Caesens et al., 2017; Bell and Khoury, 2025). We therefore propose that organizational dehumanization mediates the relationship between PAE and job satisfaction.

JD-R theory further posits that personal resources can buffer the conversion of demands into impairment outcomes (Bakker and Demerouti, 2017). We focus on proactive personality, defined as a relatively stable disposition to identify opportunities, persist in the face of constraints, and take initiative to shape one’s environment (Bateman and Crant, 1993; Seibert et al., 2001). Proactive employees are more likely to seek explanations, voice concerns, and re-craft tasks under technological constraints (Crant, 2000; Parker and Collins, 2008). We argue that proactive personality buffers the first stage of the proposed mediation, weakening the link between PAE and organizational dehumanization. This moderated mediation prediction (Hayes, 2018) specifies who is most vulnerable to dehumanization under algorithmic evaluation and provides a finer-grained answer than would the main effect alone.

We test this model with three-wave time-lagged data from 223 financial-sector employees aged 45 years or older drawn from 12 firms across five major economic regions in China (Beijing, Shanghai, Zhejiang, Jiangsu, and Guangdong). Older employees represent a theoretically informative case for several reasons. With delayed retirement and an aging workforce, mid- and late-career employees remain central to knowledge-intensive sectors yet are simultaneously stereotyped as less digitally fluent (Truxillo et al., 2015). At the same time, the financial industry has experienced rapid digital transformation that has institutionalized algorithmic risk monitoring, automated KPI tracking, and AI-augmented HR systems (Budhwar et al., 2023; Meijerink and Bondarouk, 2023). The intersection of career stage, sectoral pressure, and algorithmic intensification creates a context in which PAE is likely to be particularly salient and in which dehumanization concerns are likely to be acute, yet has been largely overlooked by extant research that concentrates on younger gig workers (Caza et al., 2022; Wood et al., 2019).

**Figure 1:**
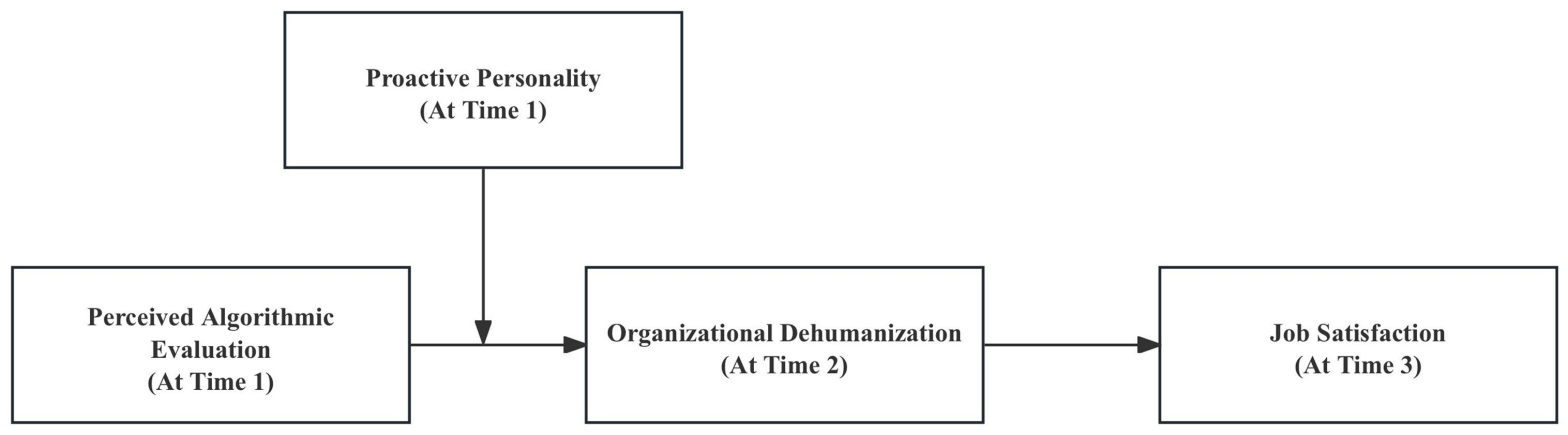
Conceptual model of moderated mediation. The conceptual model depicts a first-stage moderated mediation across three time points (T1, T2, and T3). In the model, perceived algorithmic evaluation (Time 1) is the independent variable, organizational dehumanization (Time 2) is the mediator, job satisfaction (Time 3) is the dependent variable, and proactive personality (Time 1) is the moderator of the first-stage (perceived algorithmic evaluation → organizational dehumanization) path.

Our study makes three contributions. First, we extend research on PAE, originally developed for app-workers (Zhang et al., 2025), to traditional organizational employees, demonstrating that the construct retains explanatory power outside of platform-based gig contexts. Second, by introducing organizational dehumanization as the connecting mechanism between PAE and job satisfaction, we shift attention from energetic demands (e.g., overload) to symbolic-relational demands, contributing a dignity-grounded mediator to JD-R applications of digital HRM (Bakker and Demerouti, 2017; Christoff, 2014). Third, we identify proactive personality as a personal resource, specifying boundary conditions for who is protected from algorithmic dehumanization. The conceptual model is presented in Figure 1.

## 2 Theory and hypotheses

### 2.1 Perceived algorithmic evaluation in traditional organizations

Algorithmic evaluation refers to the assessment of work attitudes, behaviors, and outcomes through real-time, algorithm-driven processes that include matching, recording, and rating (Kellogg et al., 2020; Möhlmann et al., 2021). Whereas Zhang et al. (2025) focused on app-workers’ subjective perception of these processes, coining the term perceived algorithmic evaluation, we contend that the construct is meaningful for traditional employees as well. In financial institutions, for instance, branch employees’ daily transactions, sales conversions, customer service ratings, and even compliance behaviors are increasingly captured, scored, and ranked by integrated information systems (Kellogg et al., 2020; Meijerink and Bondarouk, 2023). Although these employees occupy formal positions and enjoy long-term contracts, they nonetheless develop subjective beliefs about the extent to which algorithms judge their work. Following Zhang et al. (2025), we define PAE as employees’ perception that their work is assessed through real-time, algorithm-driven processes of matching, recording, and rating originating from the organization or its stakeholders.

Perceived algorithmic evaluation is conceptually related to, but distinct from, several adjacent constructs. Electronic performance monitoring refers to the technology-enabled collection of data about employee activities, emphasizing observation and data capture rather than the evaluative scoring of performance (Ravid et al., 2020). Algorithmic control denotes the use of algorithms to direct, evaluate, and discipline workers as a mechanism of organizational control, a broad governance construct of which evaluation is only one element (Kellogg et al., 2020). Digital surveillance foregrounds the monitoring and privacy-intrusion aspects of workplace technology, whereas HR analytics concerns the organization-level use of data to inform managerial decision-making and is typically invisible to employees (Marler and Boudreau, 2017). Perceived algorithmic evaluation differs from all of these in being an employee-level subjective appraisal specifically of being assessed—matched, recorded, and rated—by algorithmic processes. It is the experienced evaluative dimension, rather than data capture, control, surveillance, or back-end analytics per se, that we theorize as a socio-cognitive job demand with implications for dehumanization.

From a JD-R perspective, PAE can be conceptualized as a socio-cognitive job demand. Job demands are physical, social, organizational, or technological aspects of the job that require sustained effort and are associated with physiological and psychological costs (Bakker and Demerouti, 2017). PAE meets this definition along three dimensions: it heightens visibility (employees know they are watched), it imposes comparability (their performance is ranked), and it externalizes accountability (the algorithm, rather than a relational manager, defines what good performance is) (Kellogg et al., 2020; Parent-Rocheleau and Parker, 2022). These three features collectively make PAE a recurrent demand that consumes regulatory resources and can lead employees to question how they are being valued by their organization.

### 2.2 Organizational dehumanization as a mediator

Organizational dehumanization refers to employees’ subjective experience that the organization treats them as instruments, objects, or interchangeable parts rather than as full persons (Caesens et al., 2017). Recent reviews and empirical evidence link dehumanization to a range of attitudinal and well-being outcomes, including lower job satisfaction, reduced affective commitment, emotional exhaustion, and turnover intentions (Bell and Khoury, 2025; Brison et al., 2022; Caesens et al., 2017). Theoretically, organizational dehumanization captures a distinctive symbolic threat: it concerns whether one is recognized as a moral subject at work, not merely whether one is overworked or under-rewarded.

It is important to distinguish organizational dehumanization from job (dis)satisfaction, because the two constructs are conceptually and empirically separable rather than overlapping. Organizational dehumanization is a relational, appraisal-based belief about how one is treated—specifically, the perception that the organization regards the employee as an interchangeable instrument, object, or tool rather than as a full human being (Brison et al., 2022; Caesens et al., 2017). Job satisfaction, by contrast, is a global evaluative and affective attitude that reflects how favorably an employee appraises the job as a whole (Cammann et al., 1979; Locke, 1976). The former concerns the symbolic question of whether one is recognized as a person; the latter concerns the hedonic-evaluative question of whether one likes one’s job. Dehumanization is therefore a meaning-laden antecedent that can erode satisfaction without being identical to it: an employee may remain reasonably satisfied with pay or task content while still feeling objectified, or may feel humanely treated yet dissatisfied for unrelated reasons. Consistent with this distinction, the two constructs load on separate factors in our confirmatory factor analysis and are only moderately correlated (r = -0.67), confirming that organizational dehumanization is an explanatory mechanism rather than a redundant relabeling of job dissatisfaction.

We argue that PAE is positively related to organizational dehumanization for three reasons. First, algorithmic evaluation explicitly translates work into quantifiable indicators that can be compared, ranked, and replaced; this signals to employees that their value lies in their measurable contribution rather than in their unique person (Christoff, 2014; Raisch and Krakowski, 2021). Second, algorithmic evaluation often operates through opaque rules that resist relational explanation, weakening the social exchange that humanizes the employee–organization relationship (Caesens et al., 2017; Wiener et al., 2021). Third, in traditional organizations such as banks, where regulatory technology and process automation have made systematic monitoring routine, employees may interpret algorithmic evaluation as evidence that the organization prioritizes operational efficiency and risk control over individual dignity. Taken together, the more strongly employees perceive that algorithms evaluate their work, the more likely they are to feel that the organization treats them as instruments, leading to higher organizational dehumanization. This interpretation is consistent with recent evidence that employees and job applicants resist purely algorithmic evaluation and express a strong preference for retaining human involvement in assessment, precisely because automated judgment can feel impersonal and objectifying (Mirowska and Mesnet, 2022).

*Hypothesis* 1 (*H1*). Perceived algorithmic evaluation is positively related to organizational dehumanization.

Once activated, organizational dehumanization functions as a proximal driver of negative work attitudes, including reduced job satisfaction. Theoretical accounts ground this prediction in two complementary lines of reasoning. First, social exchange perspectives argue that satisfaction with the organization is sustained by reciprocal recognition: employees contribute discretionary effort and identification to the extent that they perceive the organization as valuing them as whole persons (Caesens et al., 2017; Caesens et al., 2019). When employees feel reduced to instruments, this reciprocity is breached, and the affective and relational basis of job satisfaction is undermined. Second, dignity-based accounts emphasize that work is a central domain in which adults derive a sense of moral worth and identity (Baldissarri and Fourie, 2023; Christoff, 2014). Dehumanization signals that the organization fails to acknowledge employees as moral subjects, threatening basic psychological needs for respect and belonging and, in turn, generating dissatisfaction.

Empirical evidence consistently corroborates these theoretical claims. Across a range of occupational settings, organizational dehumanization has been linked to lower job satisfaction, reduced affective commitment, increased emotional exhaustion, higher turnover intentions, and poorer subjective well-being (Bell and Khoury, 2025; Brison et al., 2022; Caesens et al., 2019; Demoulin et al., 2021). Importantly, these associations have been observed even after controlling for traditional demand-related variables such as workload and time pressure, suggesting that dehumanization captures a distinct, dignity-based pathway. Within the JD-R framework (Bakker et al., 2023; Bakker and Demerouti, 2017), this pathway represents a health-impairment-style process in which a socio-cognitive demand (PAE) initiates a symbolic strain (organizational dehumanization), which in turn erodes a key positive attitudinal outcome (job satisfaction). The mediation logic implies that PAE’s negative implications for satisfaction operate principally through dehumanization rather than through a direct affective effect; in our sample, where employees occupy hierarchical positions in financial institutions and rely on long-term relational contracts, the symbolic interpretation of algorithmic evaluation is likely to be especially diagnostic of how the organization values them as persons.

*Hypothesis 2 (H2)*. Organizational dehumanization mediates the negative relationship between perceived algorithmic evaluation and job satisfaction; that is, the positive perceived algorithmic evaluation–dehumanization association posited in H1 carries forward, through heightened dehumanization, to lower job satisfaction, because organizational dehumanization is negatively related to job satisfaction.

### 2.3 Proactive personality as a moderator

JD-R theory specifies that the impact of job demands on impairment outcomes depends jointly on the demands themselves and on the personal and contextual resources that individuals can deploy (Bakker et al., 2023; Bakker and Demerouti, 2017; Schaufeli and Taris, 2014). Personal resources are aspects of the self that promote resilience and refer to individuals’ sense of their ability to control and influence their environment effectively (Hobfoll, 2002). They function as appraisal-shaping inputs that determine whether a given job demand is encoded as a threat to be borne passively or as a challenge that can be addressed proactively (Lazarus and Folkman, 1984). Among the most extensively studied personal resources is proactive personality, a relatively stable disposition that captures the tendency to take initiative, identify opportunities, persist in the face of constraints, and bring about constructive change in the work environment (Bateman and Crant, 1993; Crant, 2000; Seibert et al., 2001). Although early conceptualizations described proactive personality as a behavioral tendency, contemporary work treats it as a person-level resource that shapes both how individuals appraise job demands and how they regulate their emotional and behavioral responses to those demands (Fuller and Marler, 2009; Parker and Collins, 2008; Thomas et al., 2010).

Building on this resource logic, we argue that proactive personality buffers the conversion of perceived algorithmic evaluation into organizational dehumanization. Three theoretically distinct mechanisms underpin this prediction. First, proactive employees are more likely to engage in active cognitive reappraisal of evaluative demands, framing algorithmic evaluation as a tool to be mastered or as feedback to be leveraged for development, rather than as an inevitable threat to their identity (Crant, 2000; Lazarus and Folkman, 1984). Reappraising the algorithm as a controllable feature of work weakens the inference that one is being treated as an interchangeable instrument. Second, proactive employees more readily seek explanations, request feedback, and propose modifications to existing rules and processes (Parker and Collins, 2008). These voice and feedback-seeking behaviors restore a sense of agency and dialogue, partially substituting the relational quality that purely algorithmic evaluation tends to suppress (Wiener et al., 2021). Third, proactive employees engage in job crafting and self-development behaviors that allow them to demonstrate value beyond what the algorithm can capture (Demerouti et al., 2014; Wrzesniewski and Dutton, 2001). By expanding their task and relational boundaries, they generate evidence to themselves and to others that they are more than the sum of their measurable outputs, mitigating the symbolic threat that PAE poses to their sense of being recognized as a person at work.

For employees lower in proactive personality, by contrast, the same level of PAE is more likely to be encoded as evidence that the organization values them only insofar as they perform on tracked indicators. Lacking the dispositional impetus to reappraise, voice, or craft, they have fewer means to neutralize the dignity-related implications of algorithmic evaluation, and consequently are more likely to translate PAE into organizational dehumanization. The age profile of our sample reinforces this expectation: older employees often hold tacit, relational, and developmental contributions that are not easily captured by quantitative metrics (Truxillo et al., 2015; Wang and Wanberg, 2017), making them particularly susceptible to the dehumanizing implications of algorithmic evaluation when they lack proactive resources to preserve a sense of being recognized as a whole person. Theoretically, this places the moderating role of proactive personality on the first stage (PAE → organizational dehumanization) rather than on the second stage (organizational dehumanization → job satisfaction): proactive resources are most consequential at the appraisal step, where the demand is interpreted as either a threat to dignity or as a manageable feature of work.

*Hypothesis 3 (H3)*. Proactive personality moderates the relationship between perceived algorithmic evaluation and organizational dehumanization, such that the positive relationship is weaker for employees with higher proactive personality.

### 2.4 Moderated mediation

Combining H2 and H3 yields a moderated mediation prediction (Hayes, 2018). Because proactive personality buffers the path from PAE to organizational dehumanization, it should also moderate the indirect effect of PAE on job satisfaction through dehumanization. Specifically, when proactive personality is higher, the buffering effect should weaken the positive PAE–dehumanization link, which in turn should attenuate the negative indirect effect of PAE on job satisfaction.

*Hypothesis 4 (H4)*. Proactive personality moderates the negative indirect effect of perceived algorithmic evaluation on job satisfaction through organizational dehumanization, such that the indirect effect is less negative when proactive personality is higher.

## 3 Method

### 3.1 Participants and procedure

Data were collected from employees aged 45 years or older working in 12 financial-services firms (commercial banks, insurance companies, and securities firms) located across five major economic regions in China: Beijing, Shanghai, Zhejiang, Jiangsu, and Guangdong. The five-region sampling frame was chosen to enhance regional representativeness, given that these regions account for a substantial share of China’s financial-sector employment and have advanced most rapidly in the adoption of algorithmic human resource management. To strengthen temporal precedence and reduce common method variance (Podsakoff et al., 2003), we adopted a three-wave time-lagged design with approximately one-month intervals between waves. With the assistance of each firm’s human resources department, 400 eligible employees were invited at Time 1 (T1) via internal email or instant-messaging channels; all completed an online questionnaire reporting perceived algorithmic evaluation, proactive personality, and demographic and work-related controls (T1 valid responses = 400). Approximately 1 month later, at Time 2 (T2), the same participants were invited to report organizational dehumanization (T2 valid responses = 320; retention from T1 = 80.0%). Approximately 1 month after T2, at Time 3 (T3), participants completed the job satisfaction measure (T3 valid responses = 256; retention from T2 = 80.0%). Responses were matched across the three waves using anonymized participant identifiers. Following Meade and Craig (2012), we additionally excluded participants who failed two or more attention-check items, who completed any wave in less than the pretest-derived minimum time, or who showed long strings of identical responses across reverse-coded items. After matching and screening, the final analytic sample comprised 223 respondents, yielding an overall completion-and-retention rate of 55.8%. Informed consent was obtained at the beginning of each wave, and participants were assured that their responses would be confidential and used solely for academic research.

To ensure that respondents were genuinely exposed to algorithmic evaluation comparable to the focal construct, participation was restricted to employees whose performance was managed through their firm’s integrated, data-driven performance-management systems. In the participating commercial banks, insurance companies, and securities firms, these systems encompass automated key-performance-indicator dashboards that track daily transactions, sales conversions, and product cross-selling; real-time scoring and ranking of customer-service quality (e.g., from call-center and customer-relationship-management records); and algorithm-assisted risk and compliance monitoring that flags employee actions against regulatory rules. Employees routinely encounter these systems in the form of algorithm-generated scores, rankings, and alerts that feed into periodic performance feedback, incentive allocation, and supervisory review. As an eligibility screen, the human-resources liaison at each firm confirmed that invited employees were currently subject to such systems, and a single-item check at Time 1 (“My work performance is recorded and evaluated by computerized/algorithmic systems”) was used to exclude any respondent who reported no exposure. Perceived algorithmic evaluation was thus assessed among employees with genuine, ongoing exposure to algorithmic evaluation.

We operationalized older (late-career) employees as those aged 45 years or older. Although chronological age has no single universal cut-off, a threshold of 45 is widely adopted in the aging-at-work and lifespan-development literatures to demarcate entry into the later career stage—the point at which age-related stereotypes about adaptability and digital fluency become salient and at which workers are increasingly categorized as “older” by employers and policymakers (Kooij et al., 2008; Ng and Feldman, 2008; Truxillo et al., 2015). This threshold is especially appropriate in the present context. Under China’s recently enacted progressive delayed-retirement reform, statutory working life now extends well beyond age 60, so employees aged 45 and older constitute a sizable and strategically important segment of the financial-sector workforce who will remain economically active for one to two additional decades. Restricting the sample to this group therefore isolates the population for whom the intersection of career stage, algorithmic intensification, and dehumanization risk is most consequential, and for whom organizational interventions are most urgent.

Sample characteristics were as follows: 69.5% were male and 30.5% were female; mean age was 53.28 years (SD = 3.15); 89.7% were married; the average years of schooling was 14.97 (SD = 1.06); and mean weekly working hours was 44.73 (SD = 6.36). Participants represented a range of functional areas, including retail banking, corporate banking, risk management, audit, and back-office operations. Ethical approval for the study was obtained from the Yiwu Industrial and Commercial College Institutional Review Board (Approval No. 2026-SFS-0405).

### 3.2 Measures

All measures were originally developed in English and were translated into Chinese using a translation–back-translation procedure (Brislin, 2016). Unless otherwise specified, items were rated on a 5-point Likert scale ranging from 1 (strongly disagree) to 5 (strongly agree). Composite scores were calculated as the mean of constituent items after appropriate reverse coding. We adopted a 5-point response format for three reasons. First, the 5-point scale is the most widely used and best-understood format in Chinese survey research; it reduces response burden and careless responding, which is particularly advantageous in a three-wave design completed by older respondents. Second, methodological evidence indicates that the number of scale points has little systematic effect on the reliability, validity, or factor structure of multi-item attitudinal measures, with 5-, 7-, and 10-point formats yielding highly comparable psychometric properties once scores are rescaled (Dawes, 2008; Krosnick and Presser, 2010; Preston and Colman, 2000). Third, holding the response format constant across all focal scales avoids method artifacts that can arise from mixing scale lengths. The acceptable reliabilities and the supported four-factor measurement model reported below indicate that the 5-point format afforded adequate variability and sensitivity for the present constructs.

### 3.3 Perceived algorithmic evaluation

Perceived algorithmic evaluation was measured at Time 1 with the four-item scale developed and validated by Zhang et al. (2025). A sample item is, “The algorithm actively monitors my progress, providing real-time, dynamic feedback.” The scale captures the three core mechanisms of algorithmic evaluation: matching, recording, and rating. Cronbach’s *α* in the present sample was 0.78.

### 3.4 Proactive personality

Proactive personality was measured at Time 1 with Seibert et al.’s (2001) 10-item adaptation of the Proactive Personality Scale (Bateman and Crant, 1993). Sample items include, “I excel at identifying opportunities,” and, “I am always looking for better ways to do things.” Cronbach’s α was 0.72.

### 3.5 Organizational dehumanization

Organizational dehumanization was measured at Time 2 with Caesens et al.’s (2017) 11-item scale, with item wording adapted to the financial-industry context. A sample item is, “If my work could be done by a machine, the organization would not hesitate to replace me with new technology.” Cronbach’s α was 0.81.

### 3.6 Job satisfaction

Job satisfaction was measured at Time 3 using the three-item global job satisfaction scale developed in the Michigan Organizational Assessment Questionnaire tradition (Cammann et al., 1979) and used by Liu et al. (2007). Items were, “In general, I like my job,” “In general, I am satisfied with my job,” and “In general, I enjoy working here.” Cronbach’s α was 0.73.

### 3.7 Control variables

Following recommendations to control for variables likely related to both predictors and outcomes (Becker et al., 2016), we included, all assessed at Time 1, gender (0 = female, 1 = male), age (in years), education (years of schooling), marital status (0 = unmarried, 1 = married), and weekly working hours. These variables have been linked to perceptions of work conditions, well-being, and attitudes in prior aging-workforce research (Truxillo et al., 2015) and to algorithmic evaluation studies (Zhang et al., 2025).

## 4 Analytical strategy

We tested the hypothesized model in three steps. First, we estimated a parcel-based four-factor confirmatory factor analysis (CFA) of PAE, proactive personality, organizational dehumanization, and job satisfaction to evaluate measurement adequacy and discriminant validity. Following Little et al. (2002), we created item-to-construct balanced parcels for the longer scales (proactive personality, organizational dehumanization), which is well suited to small to moderate samples and reduces the impact of item-level idiosyncratic variance. Because the focal scales comprise 28 items and the analytic sample contains 223 cases, parceling also yields a parsimonious measurement model with an acceptable indicator-to-sample ratio while preserving construct-level variance (Little et al., 2002). Standardized parcel loadings, composite reliabilities, AVE values, and latent factor correlations are reported in Table 1.

**Table 1:** Parcel-level standardized loadings, reliabilities, and latent factor correlations (*N* = 223).

Construct	Indicator	λ	CR	AVE
PAE	Parcel 1	0.85	0.70	0.54
	Parcel 2	0.60		
PP	Parcel 1	0.48	0.75	0.51
	Parcel 2	0.76		
	Parcel 3	0.85		
OD	Parcel 1	0.83	0.85	0.65
	Parcel 2	0.81		
	Parcel 3	0.78		
JS	Item 1	0.74	0.73	0.48
	Item 2	0.65		
	Item 3	0.68		

Latent factor correlations: PAE–PP = 0.50, PAE–OD = 0.57, PAE–JS = −0.40, PP–OD = 0.52, PP–JS = −0.50, OD–JS = −0.67. PAE, perceived algorithmic evaluation; PP, proactive personality; OD, organizational dehumanization; JS, job satisfaction; λ, standardized loading; CR, composite reliability; AVE, average variance extracted. Parcels were formed using the item-to-construct balance method (Little et al., 2002). Job satisfaction items are the three global satisfaction items.

Second, we computed descriptive statistics, internal consistency reliabilities, and zero-order correlations among focal variables and controls. Third, we tested H1–H4 using Hayes’s (2018) PROCESS Model 7 specification estimated by ordinary least squares regression with mean-centered predictors. The equation regressed organizational dehumanization on PAE, proactive personality, the PAE × proactive personality interaction, and the five controls. The second-stage equation regressed job satisfaction on PAE, organizational dehumanization, proactive personality, and the controls. Conditional indirect effects were estimated at low (M − 1 SD) and high (M + 1 SD) levels of the centered moderator. Bias-corrected bootstrap confidence intervals (5,000 resamples) were used to test the conditional indirect effects and the index of moderated mediation (Hayes, 2018).

## 5 Results

### 5.1 Confirmatory factor analysis

The hypothesized four-factor model fit the data well, χ^2^(38) = 55.93, CFI = 0.98, TLI = 0.97, RMSEA = 0.05, and clearly outperformed a three-factor model that combined perceived algorithmic evaluation and organizational dehumanization, Δχ^2^(3) = 49.59, *p* < 0.001, as well as a one-factor model, Δχ^2^(6) = 211.47, *p* < 0.001 (see Table 2). The four-factor model also yielded the lowest Akaike information criterion (AIC = 111.93, compared with 155.51 and 311.39 for the three- and one-factor models), confirming it as the preferred measurement model. Standardized parcel loadings ranged from 0.48 to 0.85; composite reliabilities ranged from 0.70 to 0.85; and average variance extracted (AVE) ranged from 0.48 to 0.65 (job satisfaction’s AVE was marginally below 0.50). The square root of each construct’s AVE exceeded its latent correlations with the other constructs, supporting discriminant validity (Fornell and Larcker, 1981; see Table 1). Together with the Cronbach’s *α* values in Table 3 (Schmitt, 1996), these results support the measurement adequacy of the four focal constructs.

**Table 2:** Confirmatory factor analysis.

Model	χ^2^	df	CFI	TLI	RMSEA	AIC	Δχ^2^
Four-factor (hypothesized)	55.93	38	0.98	0.97	0.05	111.93	—
Three-factor	105.51	41	0.92	0.90	0.08	155.51	49.59***
One-factor	267.39	44	0.74	0.67	0.15	311.39	211.47***

*N* = 223. CFI = comparative fit index; TLI = Tucker–Lewis index; RMSEA = root mean square error of approximation. Four-factor vs. three-factor: Δχ^2^(3) = 49.59, ΔAIC = 43.58. Four-factor vs. one-factor: Δχ^2^(6) = 211.47, ΔAIC = 199.46. Lower AIC indicates better fit. ***p < 0.001.

**Table 3:** Means, standard deviations, reliabilities, and correlations among study variables (*N* = 223).

Variable	M	SD	α	1	2	3	4	5	6	7	8	9
1. Gender	0.70	0.46	—	—								
2. Age	53.28	3.15	—	0.04	—							
3. Education	14.97	1.06	—	0.08	0.00	—						
4. Marital status	0.90	0.30	—	0.03	−0.20**	0.06	—					
5. Work hours	44.73	6.36	—	−0.20**	−0.16*	0.02	−0.05	—				
6. PAE	1.95	0.54	0.78	−0.03	0.10	0.08	−0.08	0.03	—			
7. PP	1.73	0.38	0.72	−0.22**	−0.11	0.05	0.04	0.21**	0.39***	—		
8. OD	2.09	0.49	0.81	−0.01	−0.10	−0.06	0.06	0.13	0.43***	0.41***	—	
9. JS	4.24	0.57	0.73	0.09	0.12	−0.00	0.01	−0.19**	−0.28***	−0.38***	−0.51***	—

PAE, perceived algorithmic evaluation; PP, proactive personality; OD, organizational dehumanization; JS, job satisfaction. Gender: 0 = female, 1 = male. Marital status: 0 = unmarried, 1 = married. Education = years of schooling. Work hours = weekly hours worked. Cronbach’s α reported for multi-item scales only. *p < 0.05. **p < 0.01. ***p < 0.001 (two-tailed).

### 5.2 Descriptive statistics and correlations

Means, standard deviations, internal consistency reliabilities, and bivariate correlations are presented in Table 3. PAE was positively correlated with organizational dehumanization (r = 0.43, *p* < 0.001) and negatively with job satisfaction (r = −0.28, *p* < 0.001). Organizational dehumanization was negatively related to job satisfaction (r = −0.51, *p* < 0.001). Proactive personality showed a complex zero-order pattern, correlating positively with PAE (r = 0.39, *p* < 0.001) and organizational dehumanization (r = 0.41, *p* < 0.001) and negatively with job satisfaction (r = −0.38, *p* < 0.001). Although these zero-order signs may at first appear to contradict the hypothesized buffering role of proactive personality, the multivariate analyses below clarify that the moderating role of proactive personality on the PAE–dehumanization link is buffering after partialling shared variance (cf. MacKinnon et al., 2000).

### 5.3 Hypothesis tests

Table 4 presents the OLS regression results for the moderated mediation model. Consistent with H1, PAE positively predicted organizational dehumanization after controlling for demographic variables, b = 0.365, SE = 0.062, t = 5.89, *p* < 0.001. Consistent with the mediation logic of H2, organizational dehumanization negatively predicted job satisfaction, b = −0.496, SE = 0.079, t = −6.28, *p* < 0.001. The direct effect of PAE on job satisfaction in the presence of the mediator was not significant, b = −0.034, SE = 0.071, *p* = 0.635, consistent with full statistical mediation through organizational dehumanization. The bias-corrected bootstrap 95% confidence interval for the average indirect effect of PAE on job satisfaction through organizational dehumanization excluded zero (see Table 5), supporting H2.

**Table 4:** Regression results for moderated mediation.

Predictor	OD: b	SE	t	*p*	JS: b	SE	t	*p*
Gender	0.068	0.063	1.08	0.282	0.043	0.074	0.58	0.561
Age	−0.013	0.009	−1.43	0.155	0.010	0.011	0.91	0.365
Education	−0.052	0.026	−1.96	0.051	−0.012	0.031	−0.38	0.707
Marital status	0.103	0.094	1.09	0.276	0.084	0.110	0.76	0.448
Work hours	0.005	0.005	1.10	0.274	−0.008	0.005	−1.45	0.149
PAE	0.365	0.062	5.89	< 0.001	−0.034	0.071	−0.48	0.635
PP	0.367	0.085	4.31	< 0.001	−0.255	0.103	−2.49	0.014
PAE × PP	−0.253	0.116	−2.19	0.030	—	—	—	—
OD	—	—	—	—	−0.496	0.079	−6.28	< 0.001
R^2^	0.302				0.316			

OD, organizational dehumanization; JS, job satisfaction; PAE, perceived algorithmic evaluation; PP, proactive personality. b, unstandardized OLS coefficient. Continuous predictors mean-centered before forming the interaction term. Controls (gender, age, education, marital status, work hours) appear in both equations.

**Table 5:** Conditional indirect effects and index of moderated mediation (Bootstrap *N* = 5,000).

Condition/index	Estimate	SE_boot	95% CI LL	95% CI UL
IE at low PP (−1 SD)	−0.231	0.057	−0.353	−0.127
IE at high PP (+1 SD)	−0.136	0.039	−0.221	−0.068
Contrast (IE_low PP − IE_high PP)	−0.095	0.038	−0.176	−0.026
Hayes IMM (θ_PAE × PP × β_OD → JS)	0.125	0.039	0.033	0.231

IE, indirect effect of perceived algorithmic evaluation on job satisfaction via organizational dehumanization. PP = proactive personality. Bootstrap CIs = bias-corrected percentile intervals (5,000 resamples). The contrast (IE_low PP − IE_high PP) is reported for transparency; the Hayes index of moderated mediation (IMM) is the formally appropriate test of moderated mediation in PROCESS Model 7. The positive IMM, combined with negative conditional indirect effects, indicates that the indirect effect becomes less negative as PP increases (buffering).

**Figure 2:**
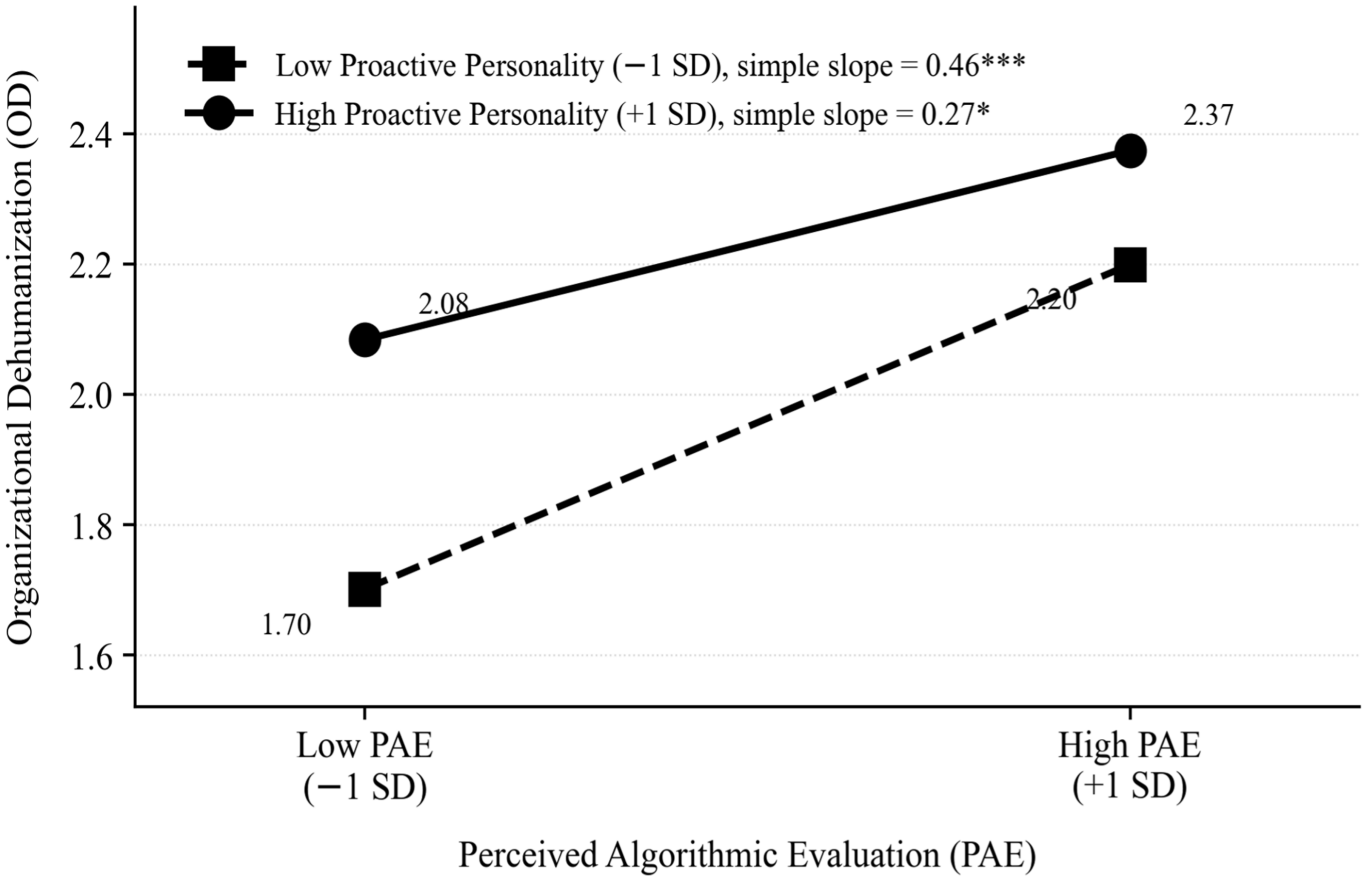
Simple slopes of perceived algorithmic evaluation predicting organizational dehumanization at low and high Levels of proactive personality. Simple slopes were estimated following Aiken and West (1991) and Dawson (2014). The PAE × PP interaction was negative and statistically significant (b = −0.253, *p* = 0.030).

H3 predicted that proactive personality would buffer the relationship between PAE and organizational dehumanization. As expected, the PAE × proactive personality interaction term was negative and significant, b = −0.253, SE = 0.116, t = −2.19, *p* = 0.030 (Table 4). Simple slopes analyses (Aiken and West, 1991) at low (M − 1 SD), mean, and high (M + 1 SD) values of proactive personality revealed that the positive slope of PAE on organizational dehumanization was steeper for employees with low proactive personality (b = 0.46, *p* < 0.001) than for those at the mean (b = 0.37, *p* < 0.001) or with high proactive personality (b = 0.27, *p* < 0.05; see Figure 2). Consistent with H3, the buffering pattern was such that PAE translated more strongly into dehumanization when proactive personality was lower.

Finally, H4 predicted that proactive personality would moderate the indirect effect of PAE on job satisfaction through organizational dehumanization. Table 5 reports the bootstrap results. The conditional indirect effect was significantly negative at low proactive personality, IE = −0.231, 95% CI [−0.353, −0.127], and at high proactive personality, IE = −0.136, 95% CI [−0.221, −0.068]. The Hayes index of moderated mediation was positive and significant, IMM = 0.125, 95% CI [0.033, 0.231]. Because both the OD → job satisfaction path coefficient and the conditional indirect effects were negative, a positive IMM indicates that the indirect effect became less negative as proactive personality increased, consistent with the buffering pattern in H3 and supporting H4. In other words, although organizational dehumanization always reduced job satisfaction, the strength of the negative indirect transmission from PAE to job satisfaction via dehumanization was attenuated for employees with higher proactive personality.

Robustness checks. To assess whether job function confounded the focal relationships, we re-estimated the first-stage model with dummy-coded job-role categories (retail banking, corporate banking, risk management, audit, back-office operations, and other functions) added as covariates. The effect of perceived algorithmic evaluation on organizational dehumanization was essentially unchanged (b = 0.39, *p < 0.001*), and the perceived algorithmic evaluation × proactive personality interaction retained the same direction and a comparable magnitude (b = −0.23); it became marginally significant (*p* = 0.061), consistent with the loss of degrees of freedom from several sparsely populated role categories. Job role therefore did not account for the observed pattern.

## 6 Discussion

Drawing on JD-R theory, this study examined whether perceived algorithmic evaluation is associated with reduced job satisfaction through organizational dehumanization, and whether proactive personality buffers this indirect process among older employees in China’s financial industry. The four hypotheses were supported. PAE was positively associated with organizational dehumanization (H1), which in turn was negatively associated with job satisfaction; the indirect effect was negative and significant (H2). Proactive personality buffered the first stage (H3), and the index of moderated mediation indicated that proactive personality attenuated the negative indirect effect (H4). These results extend research on PAE, originally developed for app-workers (Zhang et al., 2025), into a traditional, hierarchically structured occupational context populated by older employees, and refine theoretical understanding of how digital evaluation shapes attitudes through symbolic dehumanization.

### 6.1 Theoretical implications

First, our findings extend the construct of perceived algorithmic evaluation beyond the gig economy. Whereas Zhang et al. (2025) showed that PAE elicits flow experiences and superior service performance among app-workers in gamified environments, we found that, in a traditional financial-sector context with older employees, PAE is associated with adverse symbolic experiences, namely organizational dehumanization, and ultimately with reduced job satisfaction. The two sets of results are complementary rather than contradictory: gig settings emphasize challenge–skill balance and immediate digital rewards, whereas traditional settings rely on long-term identity, occupational dignity, and relational management. PAE is therefore not intrinsically beneficial or harmful; its psychological consequences depend on the broader work design and employee population. Highlighting this contextual contingency is an important corrective to literature that treats algorithmic management uniformly (Kellogg et al., 2020; Parent-Rocheleau and Parker, 2022).

Second, by introducing organizational dehumanization as a JD-R-aligned mediator, we contribute a dignity-based pathway to digitalization research. Most JD-R applications to digital work have focused on energy-related mediators such as workload, exhaustion, and burnout (Bakker and Demerouti, 2017; Demerouti et al., 2014). Our results show that PAE shapes attitudes primarily through symbolic mechanisms, that is, whether one is recognized as a person rather than only as a producer of measurable output (Caesens et al., 2017; Christoff, 2014). This dignity pathway speaks directly to recent calls to bring values, identity, and ethics back into work design (Baldissarri and Fourie, 2023; Raisch and Krakowski, 2021) and aligns with concerns that algorithmic systems may produce relational harms beyond efficiency-related ones (Wiener et al., 2021).

Third, the moderating role of proactive personality refines the JD-R proposition that personal resources buffer demands. Notably, our analyses indicate that proactive personality buffers the conversion of PAE into dehumanization rather than weakening the dehumanization–satisfaction link. Proactive employees do not necessarily enjoy higher satisfaction once dehumanized; rather, they are less likely to interpret the same algorithmic evaluation as dehumanizing in the first place. This location of the buffer is consistent with theoretical accounts that emphasize how dispositional resources shape demand appraisal (Crant, 2000; Parker and Collins, 2008) and clarifies why dispositional moderators may be more potent at the threat-appraisal stage than at the outcome stage.

A noteworthy feature of our data is that proactive personality showed unfavorable zero-order associations—correlating positively with perceived algorithmic evaluation (r = 0.39) and organizational dehumanization (r = 0.41) and negatively with job satisfaction (r = −0.38)—even though it buffered the evaluation–dehumanization link in the multivariate model. We interpret this divergence as a statistical suppression pattern rather than a substantive contradiction (MacKinnon et al., 2000). In this late-career financial-sector sample, more proactive employees appear to gravitate toward, or to be assigned to, more visible, target-driven, and heavily metricized roles, so that proactivity is positively entangled with exposure to algorithmic evaluation at the bivariate level; this shared variance carries the dehumanizing and dissatisfying signal that inflates the unfavorable zero-order correlations. Once perceived algorithmic evaluation and its interaction with proactivity are partialled out, the theoretically predicted buffering role emerges: at equivalent levels of algorithmic evaluation, more proactive employees convert that evaluation into dehumanization less strongly. Substantively, this implies that proactivity protects how a given level of algorithmic scrutiny is appraised, but does not shield employees from being exposed to such scrutiny in the first place—an important nuance for theory and for the design of organizational supports.

Fourth, our focus on older employees in finance contributes to the relatively under-developed intersection of aging-at-work and algorithmic-management research. Older employees may be particularly susceptible to dehumanization-related interpretations of algorithmic evaluation because they have accumulated relational, tacit knowledge that may not be readily captured by quantitative metrics, and because they often face stereotypes about digital fluency (Truxillo et al., 2015). Identifying proactive personality as a buffer in this population suggests that dispositional differences operate similarly across career stages, but situates the practical urgency for organizational interventions among the older workforce, who are increasingly central to delayed-retirement labor markets (Wang and Wanberg, 2017).

### 6.2 Practical implications

Because our design documents associations among perceived algorithmic evaluation, organizational dehumanization, and job satisfaction but does not directly test the efficacy of any specific intervention (e.g., transparency measures, appeal mechanisms, ethics review, or training), the recommendations below should be read as implications that follow from our theoretical interpretation rather than as empirically validated solutions.

Our findings carry several practical implications for organizations deploying algorithmic evaluation. First, given that PAE relates to job satisfaction primarily through dehumanization rather than through workload, interventions that focus solely on reducing computational complexity, system load, or working hours are likely to be insufficient. Organizations should pair algorithmic evaluation with humanization practices that explicitly affirm employees as whole persons rather than as data points. Concretely, this entails (a) transparent rule explanations that articulate, in accessible terms, what algorithms measure and how scores are produced; (b) routine human oversight of consequential decisions such as promotion, performance ratings, and disciplinary actions; and (c) employee voice channels through which workers can contest scores, propose changes, and surface concerns about evaluation criteria (Kellogg et al., 2020; Lee, 2018). Such complementary practices can buffer the dignity-related strain that algorithmic evaluation otherwise produces.

Second, because proactive employees more easily neutralize the dehumanization potential of PAE, managers should provide targeted support for employees lower in proactive personality. Practical examples include structured one-on-one explanations of algorithmic decisions, opportunities for participation in goal setting, accessible appeal mechanisms, and dyadic mentoring that helps less proactive employees cultivate voice and reappraisal skills (Newman et al., 2020). These interventions partially substitute the dispositional resources that proactive employees deploy spontaneously and prevent algorithmic evaluation from amplifying within-team disparities in well-being. Importantly, such supports should be designed in ways that do not stigmatize less proactive employees, but instead frame them as standard organizational scaffolding for working with AI.

Third, financial institutions in particular should recognize that the legitimacy of algorithmic HRM depends not only on accuracy and efficiency, but also on whether employees feel valued as people. Investments in algorithmic literacy training, ethics review of monitoring practices, and managerial training in algorithmic-feedback delivery can reduce the dehumanizing implications of digital evaluation while preserving its informational value (Glikson and Woolley, 2020; Raisch and Krakowski, 2021; Wiener et al., 2021). When supervisors are trained to translate algorithmic outputs into developmental conversations and to acknowledge the qualitative aspects of work that algorithms tend to miss (e.g., relational service, mentoring, risk judgment), employees are more likely to perceive evaluation as a fair, growth-oriented process rather than as an objectifying mechanism.

Fourth, our findings have specific implications for managing an aging workforce in digitally transforming sectors. Older employees often bring tacit knowledge, regulatory experience, and relational capital that algorithmic systems struggle to capture (Bal and De Lange, 2015; Truxillo et al., 2015; Wang and Wanberg, 2017). When these contributions are systematically under-weighted by algorithmic metrics, dehumanization risks are heightened. Organizations that intend to retain late-career employees, a strategic priority in many graying labor markets, should design evaluation systems that explicitly incorporate qualitative judgment, mentoring effort, and longitudinal contributions, and should pair algorithmic scores with structured developmental dialogue tailored to career-stage needs.

Finally, at the policy and governance level, the present findings suggest that algorithmic HRM should be subject to ongoing organizational ethics review, with monitoring of dignity-related outcomes (e.g., organizational dehumanization, sense of being treated as a person) added to existing efficiency-focused performance dashboards. Cross-functional committees that include HR, IT, employee representatives, and ethics officers can play a useful role in periodically auditing algorithmic systems for unintended psychological consequences and in proposing humanization adjustments. Such governance arrangements help ensure that the benefits of AI-augmented HR are realized without eroding the fundamental human dimension of work (Newman et al., 2020; Raisch and Krakowski, 2021).

## 7 Limitations and future research

Several limitations warrant consideration alongside corresponding directions for future research. First, although our three-wave time-lagged design with approximately one-month intervals strengthens temporal precedence and helps mitigate common method variance (Podsakoff et al., 2003), it cannot fully establish causal inference, and the chosen one-month interval may not match the natural pace at which PAE produces dehumanization or affects satisfaction. Future research could combine survey data with archival indicators of algorithm exposure (e.g., system usage logs, performance dashboards) or employ randomized field experiments and vignette studies that manipulate features of algorithmic evaluation to test its causal effects on dignity-related outcomes (Spector, 2019). Experience-sampling and daily-diary designs would also enable researchers to track within-person fluctuations in PAE and dehumanization at finer time scales.

Second, although our outcome (job satisfaction at Time 3) was temporally separated from the predictor (perceived algorithmic evaluation at Time 1), all focal variables remained self-reported, leaving residual concerns about common method variance and same-source reporting biases. The CFA evidence and the presence of a robust interaction effect, which is generally less susceptible to method bias (Siemsen et al., 2010), partially mitigate these concerns. Nevertheless, future work should employ multi-source designs that combine self-reports with supervisor-rated job satisfaction or HR-recorded behavioral indicators, including turnover, absenteeism, and customer service quality, in order to triangulate the proposed associations and rule out residual common-method artifacts. Relatedly, perceived algorithmic evaluation was assessed as a subjective perception rather than verified against objective system logs; future work should incorporate objective indicators of algorithm exposure and further validate the translated scales in Chinese late-career samples.

Third, the present sample, although informative, is restricted to older employees in China’s financial sector and may not generalize to younger employees, other industries, or non-Chinese contexts. The mid- to late-career stage examined here may be systematically more sensitive to dehumanization-related interpretations of algorithmic evaluation than younger career stages (Truxillo et al., 2015; Wang and Wanberg, 2017), and Chinese organizational culture, with its strong emphasis on relational ties and managerial discretion, may either amplify or dampen the symbolic interpretations under study (Caesens et al., 2017). Cross-cultural and cross-industry replications are warranted, especially in contexts where algorithmic evaluation is a more recent introduction. Comparative designs that contrast older and younger employees within the same organization would also help isolate age-graded mechanisms. Future studies could also broaden the design by sampling additional industries and by adding younger employees as a comparison group, enabling a direct cross-cohort and cross-sector comparative analysis of how PAE translates into dehumanization and dissatisfaction.

Fourth, our model focuses on a single personal resource (proactive personality). Other personal resources, including general self-efficacy, psychological capital, occupational future time perspective, and growth mindset, may operate in parallel or interactively (Hobfoll, 2002; Schaufeli and Taris, 2014). Contextual resources, such as perceived organizational support, supervisor support, leader algorithmic-feedback skill, team psychological safety, and procedural justice climate, may also moderate the path from PAE to organizational dehumanization at higher levels of analysis (Caesens et al., 2017; Parent-Rocheleau and Parker, 2022). Future research could test multi-resource models that examine which resources are most diagnostic of dehumanization risk and how they combine to buffer algorithmic strain.

Fifth, system-level features of algorithmic evaluation, beyond employees’ subjective perception of being scored, deserve further attention. Algorithmic transparency, explainability, accuracy, opportunity for human override, and procedural fairness in design are all candidate moderators that may attenuate or amplify the link between PAE and organizational dehumanization (Lee, 2018; Newman et al., 2020; Parent-Rocheleau and Parker, 2022). Future studies could integrate survey data with objective system-design audits and experimental manipulations of algorithmic features to disentangle which design choices are most consequential for dignity-related outcomes.

Sixth, although we focused on job satisfaction, the dignity-based pathway implicated by our findings is likely to extend to a broader array of attitudinal, behavioral, and well-being outcomes. Future research should examine whether organizational dehumanization mediates the effect of PAE on affective commitment, turnover intentions, organizational citizenship behavior, counterproductive work behavior, occupational health (e.g., burnout, depressive symptoms, sleep quality), and innovative behavior (Bell and Khoury, 2025; Demoulin et al., 2021). Examining these outcomes simultaneously would help establish the breadth of the dehumanization mechanism and identify potentially compensatory effects of proactive personality across domains.

## 8 Conclusion

Algorithmic evaluation is reshaping work in traditional organizations, including the lives of older employees who have long relied on relational, identity-based forms of recognition. Our results suggest that, when employees perceive that algorithms evaluate their performance, they are more likely to feel that the organization treats them as instruments rather than as persons, a symbolic experience that erodes job satisfaction. Proactive personality acts as a personal resource that buffers this conversion at the first stage. As organizations continue to integrate algorithmic systems into their HRM functions, attending to the dignity-related implications of evaluation, and supporting employees who lack dispositional buffers, will be central to maintaining satisfied, engaged, and humanly valued workforces.

## Data Availability

The raw data supporting the conclusions of this article will be made available by the authors, without undue reservation.
